# Establishing a minimum data set for suicide and attempted suicide registry system in Iran

**DOI:** 10.1186/s12889-022-13276-9

**Published:** 2022-04-29

**Authors:** Mohsen Shafiee, Mostafa Shanbehzadeh, Hadi Kazemi-Arpanahi

**Affiliations:** 1 Department of Nursing, Abadan University of Medical Sciences, Abadan, Iran; 2 Department of Health Information Technology, Ilam University of Medical Sciences, Ilam, Iran; 3 Department of Health Information Technology, Abadan University of Medical Sciences, Abadan, Iran; 4 Department of Student Research Committee, Abadan University of Medical Sciences, Abadan, Iran

**Keywords:** Suicide, Attempted, Common data elements, Registries, Risk factors, Data collection

## Abstract

**Background:**

Suicidal behavior is a major cause of mortality and disability worldwide. Accurate and consistent collection of data on suicide, suicide ideation, and suicide attempts presents many challenges for public health practitioners, policymakers, and researchers. This study aimed to establish a minimum data set (MDS) for integrating data across suicide registries and other data sources.

**Methods:**

The MDS proposed in this study was developed in two-stepwise stages. First, an extensive literature review was performed in order to identify the potential data items. Then, we conducted a two-round Delphi stage to reach a consensus among experts regarding essential data items and a supplementary one-round Delphi stage for validating the content of the final MDS by calculating the individual item content validity index (CVI) and content validity ratio (CVR) and using other statistical tests.

**Results:**

After the literature review, 189 data items were extracted and sent to a panel of experts in the form of a questionnaire. In the Delphi stage and CVI calculation, 55 and 10 experts participated in kappa and CVR calculation, respectively. Finally, the MDS of the suicide registry was finalized with 84 data elements that were classified into four categories, including patient profile, socio-economic status, clinical and psychopathological status, and suicide circumstances.

**Conclusions:**

The suicide MDS can become a standardized and consistent infrastructure for meaningful evaluations, reporting, and benchmarking of suicidal behaviors across regions and countries. We hope this MDS will facilitate epidemiological surveys and support policymakers by providing higher quality data capture to guide clinical practice and improve patient-centered outcomes.

## Introduction

Suicide is a major and ongoing physical-social public health concern [1]. Currently, in most countries of the world, it is considered a subset of intentional events and a set of events due to violence. This tragic phenomenon is defined as a deliberate act committed with the intent to kill oneself [2]. According to the report by the World Health Organization (WHO), each year approximately 800,000 individuals worldwide die by suicide [3]. On average, 132 suicides happen every day, i.e., more than one individual every 40 s. It is the second leading cause of mortality among those aged 15 to 29 years old globally [4, 5]. In Iran, a 20-year trend revealed worsened suicidal mortalities with an expected average rate of 9.9 per 100,000 individuals annually [6, 7].

Most suicides happen in low-and-middle-income countries (LMIC), where early identification is difficult due to shortages and restricted resources and services, as well as inadequate treatment and support [8]. However, these statistics are only the tip of the iceberg, and suicide is often underreported. Estimates indicate that suicide tolls are underestimated by 20–25% or more [9]. In addition to the suicide stigma, taking into account ethical, cultural, socio-political, and religious aspects, ineffective or non-existent documentation systems have led to the underreporting of suicides [10, 11]. Suicide documentation is a complicated and multilevel process that involves medical and legal issues, as well as responsible authorities that vary across countries [12, 13].

The suicide data are collected from a variety of data sources, and intra-sectoral collaborations are involved in this process. But, these data are not consistent and coherent; the reports on suicidal behaviors in Iran demonstrated a wide range of data collection approaches and reported tolls of suicidal behaviors [14, 15]. This inconsistency also makes it difficult to precisely trace suicide trends, demographic patterns, and time-based variations of suicide methods in populations, and thus adversely affects the development and assessment of suicide prediction plans at various levels [16]. The lack of consistent data on suicide and attempted suicides is a significant obstacle to determining the efficiency of suicide preventive plans [12]. Therefore, there is a need for more unified national suicide documentation systems to record, collect, and process data about suicidal behaviors, as well as standardized information systems related to suicide on a nationwide basis to make them findable, accessible, interoperable, and reusable (FAIR) [14]. Developing a clinical registry system is one of the valuable methods for systematic data collection. The Agency for Healthcare Research and Quality describes a registry as “a systematized system that applies observational study approaches to collect unified data to assess specified outcomes for a defined population according to a specific illness, condition, or exposure, and that aims to achieve one or further predetermined scientific, clinical, or policy purposes” [17]**.** Registries are well-established instruments for pursuing and reporting the epidemiologic features of an illness or clinical condition. They can be applied to collect information on disease evolution and patient subgroups, enable patient enrollment into experimental trials, and present practical evidence on the safety and cost-effectiveness of innovative treatments [18]. To leverage the opportunities of real-world data while strengthening research infrastructure, this study aimed to create a suicide registry system. The establishment of a minimum dataset (MDS) is one of the basic steps to warrant the standardization of data collection in the clinical registry system [19]. MDS provides a unified template for defining and homogenizing core data elements about a specific disease or clinical condition[20]. This study aimed to create an MDS to capture data on completed and attempted suicides in Iran.

## Methods

### Study design

In this study, the suicide MDS was developed in three steps as follows: First, a literature search was conducted to obtain a comprehensive overview of the elements related to suicide and map the existing evidence supporting the establishment of MDS. Then, the data items extracted from the search were ranked by a two-round Delphi survey along with a supplementary Delphi stage for validating the MDS content.

### Literature review

A comprehensive literature search was performed to identify the potential data elements in suicide studies, suicide reporting systems, and patients’ medical records. In this regard, first, a comprehensive literature review was conducted in scientific sources such as Web of Science, PubMed, ProQuest, Scopus, Magiran, and SID to extract the potential data items to be included in the suicide MDS. The conduct of the review process adhered to advanced search strategies (a combination of keywords, search operators, and search domains), and the result options (document type, publication date, and language) were refined. Full-text journal articles, reports, forms, and theses in Persian and English languages with publication dates ranging from 2000 to 2021 were included in the study. Any research that studied risk factors, circumstances, nature, population subgroups, and any other aspect of suicide was considered.

### Questionnaire design

The initial data elements were used as a working basis for developing a questionnaire to elicit panelists’ opinions about the essential data elements of suicide MDS. The importance of each data element for the final MDS was judged by a two-round Delphi method. The experts who participated in the survey were requested to assign a priority value to each data element to be included in MDS using a five-point Likert scale. The values ranged from one indicating “the lowest level of importance” to five representing the “highest level of importance”. The participant responses were anonymous throughout the survey. Finally, they were asked to propose new items that were not listed in the initial dataset for subsequent prioritization. The content validity of the questionnaire was evaluated by an expert panel, including instrument developer experts (two), psychologists (two), health information management experts (two), psychotherapists (two), and epidemiologists (two). In addition, a test–retest was used to assess the reliability of the questionnaire. A decision was made according to experts’ agreement level on data items to choose those with ≥ 75% participant agreement (e.g., regarding an item’s importance).

### Selection of a panel of experts

In the Delphi study, there is no specific method for determining the sample size, but sample size can be determined based on homogeneity, study time, extension range, availability of specialists, and study purpose. In this study, we had a homogenous sample of experts who dealt with people who committed suicide or attempted suicide. In Delphi studies, when the group of experts is homogeneous, the recommended sample size in different studies is 10–15 individuals; however, we identified 55 people based on the available experts to reduce the error rate. To select the specialists, we considered the following:

1. First, the related disciplines according to the purpose of the study should be identified.
2. Specialists in any field must have more than two years of work experience, have a related academic degree, and if possible have related scientific publishing and professional working experience.
3. Specialists should return the answers to the researchers (if any questionnaire is not returned, the participant should be excluded from the study).

### Delphi phases

An improved Delphi course was employed to reach an agreement. The survey began with participant satisfaction where each participant received an electronic suicide questionnaire to evaluate, offer recommendations, and poll in order to select important items. The important items were selected as below:

After primary ranking, data elements with less than 60% agreement were removed; those with more than 75% agreement were excluded from the next round, and those with 60% to 75% agreement were plotted in the second round. The questionnaire was separately presented to the respondents who were blind to each other’s scores, and if there was at least 75% agreement over an item, it was included in the final MDS.

### Content validity index (CVI)

In this step, we requested the panelists to give their opinions on the data elements to be included in the suicide registry MDS. The CVI was measured for all individual items (I-CVI) and the overall scale (S-CVI) [21]. To calculate CVI, the panelists were requested to rate each data element based on its importance perceived by them to be captured in the suicide registry. To sidestep a neutral opinion, a four-point Likert scale ranging from 1 to 4 (1 = not important, 2 = somewhat important, 3 = quite important, and 4 = highly important) was used. For each data element, I-CVI was calculated as the number of respondents giving a score of 3 or 4 divided by the total number of respondents. I-CVI should not be less than 0.78. One concern raised regarding the CVI is that it is an index of the interrater contract that simply expresses the proportion of the contract, and the contract can be overstated by chance factors. For this reason, the S-CVI was calculated to warrant the content validity of the overall scale. It can be conceptualized in two ways: S-CVI (universal agreement) and (average). It is suggested that the lowest S-CVI to reflect content validity is 0.8 [22].

### Statistical tests

Using the Statistical Package for Social Sciences (SPSS) software version 25 (Chicago, USA), all descriptive and analytical tests (chi-square, t-test, and paired t-test) were performed. The software was used to summarize participants’ features and demographic data. For each item outcome, the median, mean, and proportion ratings were calculated. To rank the scores, the median for each item outcome was considered. The statistical significance was set at a *p*-value < 0.05.

CVI is widely employed by investigators for specifying content validity. Nevertheless, it does not reflect the overstated values that may happen due to the likelihood of a chance contract. Kappa statistic coefficient is an agreement index of interrater agreement that was used in our study as a supplement of CVI to warrant that the promise between specialists is beyond chance. Assessment metrics for kappa statistics are that values more than 0.74, ranging from 0.6 to 0.74, and ranging from 0.4 to 0.59 are labeled as excellent, good, and fair, respectively [22, 23]. We also employed the content validity ratio (CVR) to determine whether an element is essential for inclusion in the MDS or not. For this, we have asked the panelists to score items with a three-point Likert scale ranging from 1 to 3 representing “essential”, “useful but not essential”, and “not necessary”. The numeric value of CVR ranges from -1 to 1. High scores of CVR show the contract of panel participants on the need for an element in the MDS. A positive CVR specifies that at least 50% of the panel experts agree on the need for the element to be included in the MDS [24, 25].

## Results

In this study, after searching in scientific databases and studying suicide documentation, a set of data elements was extracted and validated via a two-round Delphi survey and another one-round survey performed to calculate the I-CVI.

The questionnaire was sent through email or in person, along with a letter of request, which included the study aims, a ranking scale, and essential instructions for respondents. If no response was received for the reminder email within a week, a phone call was made or a meeting was scheduled. In the absence of any feedback, the participant was replaced.

The number of participants in the Delphi stage was 55 individuals who were selected using purposive sampling. The selection criteria for study participants included research interest in topics related to suicide conditions and having at least two years of work experience and an academic degree related to suicide and health information technology fields. About 49.09% of the contributors were female, and 85.46% of them had more than 10 years of working experience. The mean age of participants in this study and mean years of their work experience in clinical settings were 36.4 (± 5.4 SD) and 14.66 (± 4.5 SD), respectively.

In the first stage of the survey, the response rate of the experts was 100% received. In the first round of Delphi, after the application of the decision rules on the 189 extracted items, 68 items were rejected and 40 items received scores between 60 and 75%. The 40 remaining data elements were entered into the second round of Delphi. During this round, 10 items remained. The Wilcoxon test and Bonferroni correction tests were used to ensure and reduce the unknown and unwanted errors in the response rate of different expert groups in the Delphi phase. The statistical results were acceptable and the approved items in the Delphi phase were selected to calculate the CVI. After performing the Wilcoxon test and Bonferroni correction tests, distributions of the answer rates were well-adjusted among the various medical fields. After a two-round Delphi, 91 approved items were sent to an expert’s panel to determine the CVI of the items in the electronic questionnaire platform (Table 1). In this stage, we asked the experts panel to score each element based on its relevance to the suicide registry. Using a four-point scale, the I-CVI was calculated. Finally, after the Delphi survey and the calculation of CVI, 84 items were accepted for the final MDS (Table 1). Computational procedures for the scale-level CVI, which we denoted for the sake of clarity as to the S-CVI, have been completely explained in terms of scores given by 55 experts. S-CVI (universal agreement) and S-CVI (average) were calculated for each domain, and their clarity was assessed for each domain. Table 2 shows the S-CVI calculation for the patient profile domain as an example.

**Table 1:** Sample of the Delphi phase and calculation of I-CVI for the initial suicide MDS

Patient profile									
Items	Delphi phase						*Calculation of I-CVI*		Final Decision
	Round 1			Round 2					
	Agree N (%)	Dis agree N (%)	Unsure N (%)	Agree N (%)	Dis agree N (%)	Unsure N (%)	Relevant (Rating 3 or 4)	I-CVIs	
Gender	100	0	0				55	1	Kept
Age	100	0	0				55	1	Kept
Birthdate	89.23%	9%	1.77%				49	0.89	Kept
Marital status	100%	0	0				55	1	Kept
Occupation/Job	95.23%	1%	3.77%				52	0.95	Kept
Residence	92.23%	2.36%	5.41%				51	0.92	Kept
Education level	98.23%	0	1.77				54	0.98	Kept
Racial status	85.65	12.36	1.99				44	0.8	Kept
Visits followed by	91.23%	8.23	1.08%				49	0.89	Kept
Discharge deposition	89.32%	9.12	1.56				45	0.81	Kept
Healthcare setting name	54%	43.2%	2.8%						Removed
Visit type	78%	18.23%	3.77%	58.12%	41.88%	0	32	0.58	Removed
Ward admission	74.68%	21.12%	4.2%	78.36%	20.1%	1.54%	30	0.54	Removed
Physician admission	68%	30.2%	1.8%	75.2%	21.32%	3.48%	32	0.58	Removed
Referral institute	61.23%	33.23%	5.54%	76.32%	23.68%	0	32	0.58	Removed
**Socio-economic factors**									
Religion	95.63%	2.4%	0				53	0.97	Kept
Religious commitment	99.87%	0.13%	0				55	1	Kept
Primary caregiver cohabiting with partner	98.6%	1.4%	0				54	0.98	Kept
Family conflict	69.43%	29.11%	1.46%	85.46%	14.54%	0	44	0.8	Kept
Peer conflict	100	0	0				55	1	Kept
Spouse problems	92.87%	7.13%	0				53	0.97	Kept
Relationship breakdown with an intimate partner (past 1 month)	90.56%	7.4%	2.04%				52	0.95	Kept
Legal issues	99.1%	0.9	0				55	1	Kept
Prison	85%	9.23%	5.77%				49	0.89	Kept
Death of a close family	88.12%	11.88%	4.96				51	0.92	Kept
Parental supervision	79.78%	15.26%	3%				44	0.8	Kept
Parent separation	88.12%	11.88%	4.96				44	0.8	Kept
Class social	89%	10%	1%				48	0.87	Kept
Live alone	90.39%	0	9.61%				51	0.92	Kept
Abuse	78.85%	20	1.15%				44	0.8	Kept
Lifetime abuse	98.5%	1.5%	0				55	1	Kept
Position in the household	100	0	0				55	1	Kept
Place in Household	74.23%	25.77%	0%	80.23%	18.69%	1.08%	44	0.8	Kept
Family structure	100	0	0				55	1	Kept
Family size	98.92	0	1.08				54	0.98	Kept
Social and teamwork activities	88.23%	10.11%	1.67%				48	0.87	Kept
Antisocial activities	100	0	0				55	1	Kept
Marital-partner relationship difficulties	100	0	0				55	1	Kept
Problems with family relationship	100	0	0				55	1	Kept
Acculturation	98.5%	1.5%	0				55	1	Kept
Certain attitudes	98.5%	1.5%	0				55	1	Kept
Income status	98.5%	1.5%	0				55	1	Kept
Work problems	87.25%	10.3%	2.45%				48	0.87	Kept
Level of socioeconomic welfare	100%	0	0				55	1	Kept
Recent job loss	89.36	7.25	3.39				50	0.9	Kept
**Circumstances of suicide factors**									
Time of day	100%	0	0				55	1	Kept
Time of month	87.25%	10.3%	2.45%				48	0.87	Kept
Season	100%	0	0				55	1	Kept
Recent job loss	92.21%	7.79%	0				49	0.89	Kept
Place of the suicide act	73.25%	17.14%	9.61%	73.26%	18.75%	8	44	0.8	Kept
Suicide Method	100%	0	0				55	1	Kept
Motive of Suicide	100%	0	0				55	1	Kept
Type of expression of suicidal intent	89.36	7.36	3.28				50	0.9	Kept
Status of committing suicide	88.23%	10.11%	1.67%				48	0.87	Kept

**Table 2:** Modified kappa and comprehensiveness of suicide MDS

Modified Kappa and comprehensiveness of MDS							
**Items of patient profile of suicide MDS**	**The number giving a rating of 3 or 4 to the relevancy of the item**	**CVR**	**PC**^**a**^	**K**^**b**^	**Interpretation**	**The comprehensiveness of MDS dimensions**	
						**Agree**	**Proportion of consensus**
1	9	0.8	0/000858307	1	Excellent	Agreement on total comprehensiveness = 10 The comprehensiveness of entire instrument = 1	
2	10	1	0/000976563	1	Excellent		
3	10	1	0/000976563	0/889892	Excellent		
4	9	0.8	0/009765625	1	Excellent	S-CVI/Ave = 0.949 S-CVI/UN = 0.45	
5	10	1	0/000976563	0/949951124	Excellent		
6	9	0.8	0/009765625	0/919211	Excellent		
7	9	0.8	0/009765625	0/979803	Excellent		
8	8	0.8	0/043945313	0/790807	Excellent		
9	10	1	0/000976563	0/889892	Excellent		
10	9	0.8	0/009765625	0/808126	Excellent		

^a^PC (probability of a chance occurrence) was computed using the formula: pc = [N! /A! (N -A)!] *.5Nwhere N = number of experts and A = number of panelists who agree that the item is relevant

^b^K (Modified Kappa) was computed using the formula: K = (I-CVI- PC)/ (1- PC). Interpretation criteria for Kappa, using guidelines described in Cicchetti and Sparrow (1981): Fair = K of 0.40 to 0.59; Good = K of 0.60 to 0.74; and Excellent = K > 0.7

After the Delphi phase and CVI calculation, to conduct quantitative and qualitative analysis on the final suicide MDS items, a panel of 10 experts was selected. The final and approved suicide MDS after the Delphi phase and CVI calculation was sent to be judged by the panel experts, including two instrument developers, three psychologists, two psychotherapists, one health information management expert, and two epidemiologists for quantitative and qualitative analysis. The panel experts were invited to decide on CVR and MDS inclusiveness. In each phase, they also were asked to decide on the face validity of the MDS. The items of the final and approved suicide MDS were revised based on the opinions of the panel experts in the first stage of CVR and for a second stage to determine MDS modification. To determine the MDS modification, kappa was calculated to reduce the chance of error. Table 2 shows the calculation of CVR and modified kappa for items in the MDS.

Finally, the suicide MDS included 84 items that were classified into four categories (Tables 3, 4, 5 and 6).

**Table 3:** Patient profile

Data items	Content definition
Gender	Male
	Female
Age	
Birthdate	
Marital status	Married
	Single
	Divorced
	Widow
	Other, unspecified
Occupation	
Residence	Urban
	Rural
Education level	Illiterate
	Elementary
	High school
	University
Racial status	Lor
	Kurd
	Turkish
	Fars
	Other
Visits followed by	Suicide ideation
	Suicide attempt
	Intentional self-harm
	Self-harm with undetermined intent
	Death from intentional self-harm
	Death from self-harm of undetermined intent
Discharge deposition	Discharge to home
	Discharge to psycho facility
	Discharge to another facility
	Died
	other

**Table 4:** Socio-economic factors

Data items	Content definition
Religion	
Religious commitment	
Primary caregiver cohabiting with partner	
Family conflict	
Peer conflict	
Spouse problems	
Relationship breakdown with an intimate partner (past 1 month)	
Legal issues	
Prison	Pending
	Recent release
Death of a close family	
Parental supervision	Low
	Moderate
	Sever
Parent separation	
Class social	low class
	Middle class
	High class
Live alone	
Abuse	
Lifetime abuse	
Position in the household	Headman
	Child
	Wife
	Unknown
Place in household	Living with Parents
	Living Alone
	Partner Non-Married Couple without Children
	Member of Institutional Household
	other
Family structure	Nuclear family
	Extended family
Family size	2 ≥
	3–4
	≥ 4
Social and teamwork activities	Very minimum
	Minimum
	Moderate
	Much
Antisocial activities	
Marital-partner relationship difficulties	
Problems with family relationship	
Acculturation	
Certain attitudes	Authoritarianism
	Post-materialism
	Fatalism
	Stress resilience
	Traditional
	Religious
	Permissive attitude
Income status	Poor
	Medium
	Good
Work problems	
Level of socioeconomic welfare	low class
	middle class
	High class
Recent job loss

**Table 5:** Clinical or psychopathology factors

Data items	Content definition	
Present illness	Psychology disorders	Substance use disorders, schizophrenia spectrum disorders, bipolar disorder, major depressive disorder, anxiety disorders, borderline personality disorder, attention-deficit/hyperactivity disorder, autism, mood disorder, Agitation, History of Deliberate self-harm (DSH), Cognitive disorders, eating disorders, Learning disabilities, others
	Physical health problem	Acquired disorders
		Hereditary
	Mental disorder	Acquired disorders
		Hereditary
Suicidal ideation	Past	
	Current	
History of suicide attempts	Yes, No	
The intensity of suicidal ideation	Yes, No	
History of chronic disease	Yes, No	
(If yes) disease name		
Serious physical illness	Yes, No	
Serious physical illness (if yes specified)		
Felt depressed	Minor, Major, No depression	
Drug history		
Lifetime stressful (stressful events)	Yes, No	
Type of stressful event (if yes)		
Lifetime psychotic events	Yes, No	
Melancholic features	Yes, No	
Lack of confidantes	Yes, No	
Self-harm (past year)	Yes, No	
Family history of suicide attempt	Yes, No	
Mental illness/suicide in a family	Yes, No	
History of Mental illness	Yes, No	
Habitual poor coping	Yes, No	
Sleep disorder	Yes, No	
Unsatisfied with life	Yes, No	
Guilt Yes, No		
Sexual orientation	Heterosexual	
	Lesbian/gay	
	Bisexual	
	Questioning	
History of forced sexual intercourse		
Bullying victimization	Not bullied	
	School bullying	
	Cyberbullying	
	Both school and cyberbullying	
Substance dependence		
Cigarette smoking	None-smoker	
	Past smoker	
	Current smoker	
Alcohol consumption	Never	
	Current	
	Previous	
Suicide ideation	Yes, No	
Suicide attempts	Yes, No	
Ongoing interpersonal conflict	Yes, No	
Domestic violence	Yes, No	
Confusion about duty	Yes, No	
Loose camaraderie at work	Yes, No

**Table 6:** Circumstances of suicide factors

Data items	Content definition	
Date of suicide or suicide attempt		
Time of day	Morning	
	Noon	
	Evening	
	Night	
Day of week		
Month of suicide		
Time of month	First half	
	Second half	
Season	Spring	
	Summer	
	Autumn	
	Winter	
Place of the suicide act	Own home	
	Residential institution	
	Farm	
	Commercial building/trade service areas	
	Industrial areas	
	Street/highway	
	School	
	Polis custody	
	Graveyard	
	Unspecified place	
Suicide Method	Gunshot wound	Blunt force
		Contact with a blunt object
		Struck by projected object: bullet or other firearm projectiles
	Self-Immolation	Thermal mechanism
		Heating
		Contact with fire or flame
	Laceration/exsanguination	Piercing, penetrating force
		Cutting, tearing
	Hanging	Threats to breathing
		Mechanical threats to breathing
		Hanging
	Jump from the high place	Blunt force
		Contact with a blunt object
		Contacting static object
	Drowning	Threats to breathing
		Drowning and immersion
		Drowning/near-drowning following fall into the water
	Drug overdose	Poisoning by or exposure to chemical substances
		Poisoning by solid substances
	Carbon monoxide poisoning	Poisoning by or exposure to chemical substances
		Poisoning by gaseous substance
	Others	
Motive of Suicide	Psychic problem	
	Psychical problem	
	Economical problem	
	Family conflict	
	Educational problem	
	Addiction problem	
	Unemployed problem	
	Recent stressful life events	
Type of expression of suicidal intent	Verbal expression	
	Perpetration	
	Suicide Note	
	Rehearsal	
	None/unknown	
Status of committing suicide	Alone	
	In the presence of others	

## Discussion

In this study, we established an MDS for recording suicidal behaviors. MDS is a list of data fields that have been agreed to be mandatorily reported at the country level and is imperative to be determined in a cohesive and unified way from a scientific standpoint [26]. This study reached some key conclusions regarding what constitutes ‘important suicide or attempted suicide data’ to collect. In agreement with the scientific literature, patient medical records, suicide registries, as well surveillance systems, the suicide MDS was classified into four categories including patient profile, socio-economic status, clinical and psychopathological status, and suicide circumstances.

To fully cover deaths due to suicide and suicide attempts in Iran, data are gathered from four sources, including 1) data contained in the death registration system, 2) data available in the accidents reporting system that is especially collected for individuals with suicidal ideation who have been referred to the hospital. 3) Data collected by a health worker or disease specialist for cases of deaths due to suicide or suicide attempts, who have not been referred to the hospital, and 4) data collected about cases of deaths due to suicide and attempted suicide from private hospitals or clinics. Thus, in order to integrate these data sources, the developed MDS provides a structured and coherent framework for all organizations responsible for recording suicide-related data [15, 27].

Suicide reports of various countries and the districts in one country indicate differences in terms of demographics and suicide methods. The incidence of suicidal behavior methods along with the lethality of each method and variances according to age, sex, relationship status, and other demographic features illustrate change not only among environmental and cultural groups but also through various timespans [13, 28]. The developed MDS helps to investigate the features of suicidal behaviors in longitudinal timelines and compare the factors of suicide in various areas of Iran in order to recognize the basic features related to suicidal behaviors and also to prepare evidence-based suicide prevention and control programs.

Regarding suicide, the deployment of a registry upsurges research accessibility for people, while providing physicians/researchers with a consistent dataset required to improve research. In this context, a vast amount of data is produced each day in medical and medicolegal domains. In this huge data volume situation, what can be assembled is not a fundamental issue; the main focus should be on the depth and statistical power of the collected data to approve or reject a hypothesis and respond to specific questions [29, 30]. The expected hypothesis and queries to be addressed by an information system or clinical registry should determine the data elements that are favored, and resource accessibility should inform the scope of the data assembled to answer the anticipated questions [31]. The suicide registry will facilitate the efficient capture of precise, longitudinal, and nationwide data for suicide and suicide attempts. The developed registry will provide valued information on suicide prevalence, history of suicidal thoughts, and suicidal behaviors, which is presently an unmet necessity in Iran. Valued opportunities exist for a wide variety of epidemiological and clinical studies on suicide in Iran, and our developed MDS has the potential to become a significant tool in simplifying such investigations, which will be of relevance globally. The findings of the current study can be used to support policymakers, officials, clinicians, and community care workers in Iran.

The benefits of registry-based studies to realize suicide are numerous. For instance, registries have the potential to keep data on the entire population, the potential to investigate specific population sub-groups or low widespread events, have almost incessant timelines in longitudinal data, work with limited or insufficient data, have no sample attrition, and can access a huge sample of suicide clinical and sociodemographic information [26]. Registry-derived research allows the study of hard-to-reach individuals, such as those with severe psychological disorders or immigrants that habitually have been hard to recruit into research projects [29]. Furthermore, a registry-based study could be a valuable substitute for designing a prospective suicide cohort. For example, Danish investigators discovered the high suicide tolls observed in municipal zones and realized that this trend was rejected and even reversed when considering a range of contextual features including marital status, revenue, cultural variances, and psychiatric status [32]. More detailed information on how various suicide predictors are affected by individual features has also been gathered using a registry dataset, indicating among other things that being a parent to small children was a protecting feature in females but not in males, while joblessness and being solitary were risk factors only among males. Municipal living rises the likelihood of suicide among females but decreases the suicide risk in males [33].

Although the registry-based investigation of suicide has many benefits, it also faces numerous challenges. First, access to each registry is often time-consuming and expensive. A host of complex regulations depending on the requested data sources further challenges the process. Data sharing is even more difficult. Second, limitations with respect to possible research subjects must be considered because of the sensitive nature of the registered data on large portions of the population. Considering the ethical aspects of the project and working out well-grounded arguments that legitimize the project is an absolute necessity so as to gain approval. Finally, all research approvals are time-limited [34].

### Study strengths and limitations

Following an extensive literature review coupled with structured rounds of data collection, as well as accessing the collective wisdom of a panel of experts enabled us to develop a suicide dataset that can be used in healthcare and other related settings. Experts in this study agreed that the standardization of a suicide dataset is valuable, as it allows the consistent collection, analysis, and integration of data to pass among organizations responsible for suicide prevention. We hope our study can highlight the significance of data standardization and integration of suicide registries as a prerequisite for implementing suicide prevention and surveillance plans. In addition, it helps to improve the coordination of scientific research and practices to successfully address suicide and suicidal behaviors. However, our study method has some limitations that must be taken into consideration. First, given the unknown aspects of suicidal behaviors, further external validation is required; thus, we suggested that conducting a pilot study with a more comprehensive literature review and a larger expert panel could enrich the MDS. Recruiting a limited number of experts from a limited geographical area is another important challenge of the study. Therefore, this MDS must be assessed from the perspective of more multidisciplinary teams throughout the country. Finally, we used the Delphi agreement method to reach an agreement on the suicide MDS. This method has been demonstrated to be appropriate for the assessment of the requirements of information systems [35]. However, one of its limitations is that most opinions are marginalized.

### Implications for future studies

The current MDS offers a consistent, scientific, and valid template for assembling and reporting suicide data across health information systems (HISs). It will improve interoperability, comparability, reusability, integrity, reliability, feasibility, and quality criteria of data. The suicide MDS proposed in our study can help data integration in this domain and act as a basic level for interoperability between HISs. However, it is suggested that future studies investigate the technical issues towards interoperability in the suicide context.

## Conclusions

This study provides a fundamental effort towards constructing a national registry from an information management perspective to improve the suicide data quality criteria. Standardized suicide data collection is required to gain a more representative picture of suicide in Iran. Preparing a unique suicide registry program in Iran helps all the involved parties such as clinicians, police, and policymakers to devise a more appropriate and pervasive plan for the future.

## Data Availability

All data generated or analyzed during this study are included in this published article.

## Abbreviations

MDS: Minimum data set
CVI: Content validity index
CVR: Content validity ratio
FMO: Forensic medicine organization
MoHME: Ministry of health and medical education
FAIR: Findable, accessible, interoperable, and reusable
CVR: Content validity ratio
